# The implication of the coefficient of centrality for assessing the meaning of the mean

**DOI:** 10.3389/fpsyg.2014.01356

**Published:** 2014-11-28

**Authors:** David Trafimow

**Affiliations:** Department of Psychology, New Mexico State UniversityLas Cruces, NM, USA

**Keywords:** coefficient of variation, coefficient of centrality, meaning, standard deviation, meaning of mean

The coefficient of variation (*C_V_*) is an important and underused statistic that implies that the standard deviation has different meanings depending on the mean (Fisher, 1925; Yates, 1951; Yadav et al., 2013; Trafimow, 2014) and is computed by dividing the standard deviation by the mean $\left(C_{V}=\frac{\sigma }{\mu }\right)$. To gain a feel for how means impact the meanings of standard deviations, imagine that there are two classes and that the standard deviation of the final exam is 5 points in both of them but that the mean is 25 in Class 1 and 75 in Class 2. It follows that the coefficient of variation is 0.200 in Class 1 whereas it is 0.067 in Class 2. Thus, relative to the mean the standard deviation is thrice the size in Class 1 as in Class 2. Consequently, the actual value of the standard deviation is the same in both classes but its meaning is very different.

In keeping with this Special Issue on means, my goal is not to discuss how means influence the meanings of standard deviations (see Trafimow, 2014 for this discussion). Rather, it is to show that the coefficient of variation is a two edged sword so that if means modify the meanings of standard deviations, the reverse also is so. Standard deviations influence the meanings of means. An easy demonstration involves defining a new variable, termed the *coefficient of centrality* (*C_C_*), which is the reciprocal of the coefficient of variation and is given as Equation (1) below.

(1) $$C_{C}={\frac{1}{C}}_{V}=\frac{1}{\frac{\mu }{\sigma }}=\frac{\mu }{\sigma }$$

To gain a preliminary idea of how the coefficient of centrality works, suppose Company A makes pies with a mean of 100 per day and distributes them to a store that, on average, sells 100 of the pies per day. Attending only to means, life seems good because Company A is producing exactly the number of pies that maximizes profit but avoids the perils of overproduction. But now consider the standard deviation. Suppose that the standard deviation is 15 so that there is an approximately 16% chance that on any particular day, the factory will be short by 15 or more pies and a 16% chance that the factory will be long by 15 or more pies. Now, imagine that the company makes an innovation that reduces the standard deviation from 15 to 5. From a mean-centric point of view, the innovation might seem irrelevant because the mean remains at 100 pies per day. But the coefficient of centrality suggests otherwise as the innovation causes an increase in the coefficient from 6.67 to 20. Now the probability of underproduction by 15 or more pies decreases from 16% to 0.15% and the probability of overproduction by 15 or more pies decreases similarly. Clearly, the same mean value of 100 pies per day has different implications for underproduction and overproduction depending on the standard deviation.

Let us now consider Company B that also makes pies. This company produces a mean of 105 pies per day, with a standard deviation of 15 pies, even though their outlet is only willing to buy 100 pies per day. A possible reason for overproducing is that it is much worse to anger the customer by not having enough pies than to overproduce. Assuming this reason is valid, if we take Company A before its innovation when it also had a standard deviation of 15 but a mean of 100, it is obvious that the mean of Company B is higher than the mean of Company A, and Company A is therefore more at risk of angering its customer base. But let us now compare the means of the two companies after the Company A innovation reduced its standard deviation to 5. Which mean is larger? It depends on what we mean by “larger.” At first blush, the mean is 105 for Company B and 100 for Company A and so the mean is larger for Company B than for Company A. On the other hand, consider the coefficients of centrality; these are 7 for Company B and 20 for Company A and suggest the opposite conclusion. Which conclusion is correct? It depends on the goal. If the goal were simply to maximize pie production over a period of time, then a mean of 105 is superior to a mean of 100. But if the goal is to avoid dramatic underproduction, then the latter conclusion is correct; Company B (despite the mean of 105) will have more days of dramatic underproduction than will Company A (despite the mean of 100). The coefficient of centrality demonstrates that means have different meanings depending on standard deviations.

Consider a more basic example. One professor teaches an undergraduate class on abnormal psychology and another professor teaches an undergraduate class on cognitive psychology where the mean scores on the final exam are 75% and 65%, respectively. Is the abnormal psychology class better than the cognitive psychology class? Suppose that the standard deviations are 25% and 5% so that the coefficients of centrality are 3 and 13, respectively. Relative to the standard deviations, the cognitive psychology class mean well exceeds the abnormal psychology class mean, which suggests exactly the opposite conclusion. Of course, there are many other factors that could be at play but the coefficient of centrality suggests that it can be a mistake to consider the means without also considering the standard deviations.

In light of this example, it is worth mulling over advanced statistical literatures pertaining to standard deviation weighted analysis of variance (Kulinskaya et al., 2003) and weighted least squares linear regression (Strutz, 2010). These analyses result in means that are weighted by standard deviations to take differing standard deviations (heteroscedasticity) into account. Given the availabilities of the proposed coefficient of centrality and these advanced methods, it is difficult to justify researchers routinely failing to consider standard deviations when interpreting their means in future research, regardless of how complicated the data happen to be.

## Conflict of interest statement

The author declares that the research was conducted in the absence of any commercial or financial relationships that could be construed as a potential conflict of interest.
